# Spectro-kinetic modelling of photocatalytic oxidation of heterocyclic compounds in a continuous-flow packed bed reactor

**DOI:** 10.1039/d5ra09830k

**Published:** 2026-04-17

**Authors:** Rohit Pal, Ramin Farnood

**Affiliations:** a Department of Chemical Engineering and Applied Chemistry, University of Toronto 200 College Street Toronto ON M5S 3E5 Canada ramin.farnood@utoronto.ca

## Abstract

Mechanistically derived predictive kinetic models for photocatalytic processes have inherent challenges due to short lived charge carriers and free radical intermediate species. In this work, we present a spectro-kinetic model for the photocatalytic oxidation of carbazole (CAB), a nitrogen-containing heterocyclic compound, using a visible-light-active Ag/AgBr/TiO_2_ photocatalyst immobilized on glass beads. Experiments were performed in a continuous-flow packed bed photoreactor in industrial wastewater matrix under visible light. Reactive oxygen species (ROS) were monitored using electron paramagnetic resonance (EPR) spectroscopy, identifying hydroxyl radicals (˙OH) as the predominant ROS, with superoxide (O_2_˙^−^). and H_2_O_2_ acting as intermediates. The model incorporates key pathways including direct hole oxidation and ˙OH-mediated oxidation of CAB, as well as charge recombination and radical quenching. Quenching of (˙OH) by intermediate CAB-derived ˙CR radicals emerged as the rate-determining step, exerting the greatest impact on apparent quantum efficiency (AQE). The model showed excellent agreement with experimental results (*R*^2^ = 0.99), accurately predicting CAB degradation kinetics. Parametric analysis confirmed ˙OH radical mediated oxidation as the primary pathway, followed by secondary contribution from direct h^+^ attack. The system achieved 54% CAB removal with an AQE of 75%. This study demonstrates the value of integrating spectroscopic measurements with mechanistic modeling to guide photocatalytic process development.

## Abstract 2

Plasmonic photocatalysis in a continuous-flow reactor generates e⁻/h⁺ and ROS. EPR enables time-resolved detection of intermediates coupling mechanistic and kinetic frameworks within a spectro-kinetic model for photocatalytic process development.

## Graphical abstract

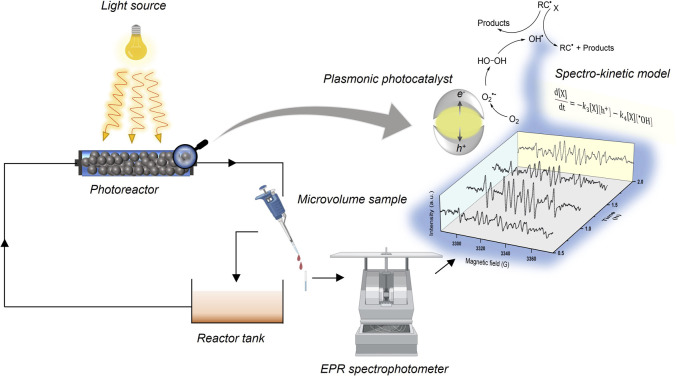

## 1. Introduction

Photocatalysis drives chemical reactions by absorbing light energy to generate electron–hole pairs (e^−^/h^+^) in photoactive semiconductor materials. These e^−^/h^+^ can produce reactive oxygen species (ROS), and/or directly attack surface adsorbed species transforming them *via* free radical-mediated redox pathways.^1–3^ Low application cost, high product yield and the unique capability to drive thermodynamically challenging bond formation have positioned photocatalysis at the forefront of diverse industrial applications, such as active pharmaceutical ingredient (API) synthesis, carbon valorization and wastewater treatment. Notably, photocatalysis has enabled C–C bond scission in structurally inert aromatic compounds *via* a single-electron transfer (SET) step at room temperature and mild conditions.^4–6^ Nitrogen containing heterocyclics are one such class of aromatic compounds which serve as the scaffold of several organic micropollutants, pharmaceutical molecules and biopolymers.^7–9^ Targeted photocatalytic reactions involving N-heterocyclic compounds such as pyridine, indole and carbazole in aqueous media have thus been a focal point of interest.^8^

Photocatalytic redox reactions rely on the use of semiconductors whose bandgap and band edge positions are suitable for absorbing light and driving the target redox processes. Titanium dioxide (TiO_2_), widely used as the photocatalyst of choice can generate ROS such as hydroxyl radicals (˙OH), singlet oxygen (^1^O_2_) and superoxide anions (O_2_˙^−^). A key limitation of using TiO_2_ is its wide bandgap (3.2 eV) which restricts its photoactivity to UV light.^10–12^ Several strategies such as forming heterojunctions of TiO_2_ with other narrow bandgap semiconductors like Cu_2_O/Au nanoparticles (2 eV), AgBr (2.6 eV) and Ta_3_N_5_ (2.1 eV) have shown promising results.^13,14^ The primary challenge with narrow band gap semiconductors such as Cu_2_O and Ta_3_N_5_ is their poor stability and positive onset potential which limit their application for solar photocatalytic application.^15,16^ AgBr, on the other hand exhibits better stability and enhanced photocatalytic effect due to surface plasmon resonance. Upon irradiation, AgBr absorbs visible light and acts as auto-catalytic centres for reduction of Ag^+^ to metallic Ag nanoparticles which exhibits surface plasmon resonance in visible light.^17,18^ Br^−^ acts as sites of electron cloud^19^ which further accelerates the e^−^/h^+^ transfer capacity. The photogenerated electrons (e^−^) in TiO_2_ recombine with the holes (h^+^) in AgBr acting as electron mediators. As a result, the h^+^ lifespan is prolonged to produce ˙OH from water or oxidize adsorbed species directly, thereby improving the photocatalytic performance and stability of Ag/AgBr/TiO_2_ heterojunction.

Most photocatalytic oxidation processes report using TiO_2_ in slurry form which leads to its agglomeration and decreased photon delivery in the reaction media at high loadings.^20–22^ These issues could be addressed by immobilizing TiO_2_ on the surface of a support material such as silica, MOF, glass beads or, zeolite.^23,24^ Synthesis methods such as sol–gel, involve series of hydrolysis and condensation reactions enabling TiO_2_ to form viscous gel which subsequently freeze on the support material.^24^ In this study, we used a modified sol–gel technique to immobilize our previously developed visible light active Ag/AgBr/TiO_2_ photocatalyst^25^ on glass beads and setup a continuous-flow packed bed photoreactor.

While several studies have focussed on material development and reactor design, research involving kinetic modeling with elemental reaction steps of intermediate species in immobilized photocatalysts has been limited. This gap has hindered the scalability and industrial application of photocatalytic technology. Existing kinetic models for photocatalytic reactions are mostly phenomenological and one-dimensional, often considering the effect of a single parameter at a time. The Langmuir–Hinshelwood^26–28^ is a common photocatalytic kinetic model, which simply considers linear or mixed linear dependence of the reaction rate on light intensity. Other factors such as humidity have been empirically introduced to this model.^29,30^ Such kinetic models have limited scope and lack generalizability. Reaction intermediates formed during photocatalytic oxidation compete with the surface adsorbed species and ROS for the same photocatalytic active sites. The e^−^/h^+^ undergo recombination before participating in SET reactions which can further reduce the photocatalytic efficiency. Therefore, to achieve scalable and predictive application of photocatalysis, it is essential to develop mechanistically informed kinetic models that incorporate elemental steps of e^−^/h^+^ lifetime and ROS generation pathways.

From a thermodynamic standpoint, if e^−^ in the CB of Ag/AgBr/TiO_2_ photocatalyst has a more negative chemical potential than the redox potentials of reactions (i)–(iii) in Table 1, then ROS form *via* SET in oxygen reduction reactions. For water oxidation, *i.e.* reaction (iv), to occur, holes in the VB Ag/AgBr/TiO_2_ photocatalyst need to lie at a more positive potential level than the water oxidation level.^31^ However, the thermodynamic drawback of a 3 e^−^ step reduction to form ROS is high compared to a single step water splitting reaction *via* h^+^.^32–34^ Thus, the actual redox pathway involved in the ROS formation is determined by kinetic pathway.^35^

**Table 1:** Redox potentials for reactive oxygen species (ROS) associated redox couples^36^

Redox reaction	Redox couple	Redox potential (V *vs.* NHE at pH 7.0)	
**Oxygen reduction reaction**			
O_2_ + e^−^ → O_2_˙^−^	O_2_/O_2_˙^−^	−0.16	(i)
O_2_˙^−^ + e^−^ + 2H^+^ → H_2_O_2_	O_2_˙^−^/H_2_O_2_	0.89	(ii)
H_2_O_2_ + e^−^ + H^+^ → H_2_O + ˙OH	H_2_O_2_/˙OH	0.38	(iii)

**Water splitting**			
H_2_O + h^+^ → H^+^+˙OH	H_2_O/˙OH	2.32	(iv)

The complexity of such multi-pathway oxidation mechanisms that occur in heterogeneous photocatalysis^37^ makes the implementation of a detailed mechanistic model evermore important. Developing mechanistic models which track ROS pathways and free radical intermediates generated upon light excitation require progress in kinetic modeling and analytical spectroscopic techniques.^37^ EPR spectroscopy has emerged as a powerful tool in the detection of ROS and other free radicals.^38^ By setting up precise experimental conditions, EPR method could quantitatively determine various reactive radical intermediates^39,40^ and predominant reaction pathways. The combination of spectroscopic tools and kinetic modeling, we could uncover the elemental steps controlling the reaction mechanism of any photocatalytic oxidation process.

In this study, we present a mechanistically derived spectro-kinetic model to describe the photocatalytic oxidation of heterocyclic compounds. Using carbazole (CAB) as the model compound, we investigate its degradation in the presence of Ag/AgBr/TiO_2_ photocatalyst under visible light irradiation. The model incorporates key reaction steps such as the competitive oxidation of model compound, generation of reactive oxygen species, and subsequent intermediates quantitation. We also explore the competition between different reaction pathways, including direct oxidation of CAB by photogenerated h^+^ and indirect oxidation mediated by ˙OH radicals. The study was conducted in a continuous flow packed bed photoreactor using glass beads to immobilize Ag/AgBr/TiO_2_ photocatalyst. Real industrial wastewater was chosen as the reaction to account for naturally occurring ROS and free radical intermediates alongside the model compound during photocatalytic oxidation.

## 2. Materials and methods

### 2.1. Immobilized Ag/AgBr/TiO_2_ photocatalyst

**Fig. 1:**
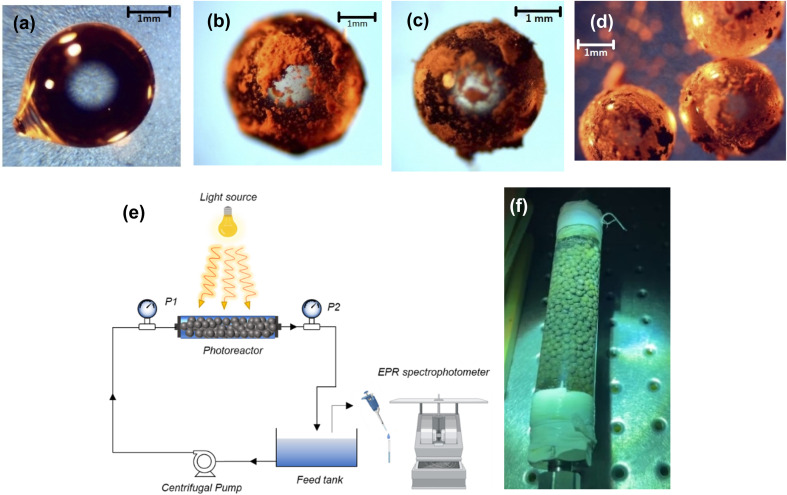
(a) Clean glass beads (b) immobilized glass beads before and, (c) after photocatalytic oxidation (d) packed immobilized glass beads, (e) schematic of continuous bench scale photocatalytic degradation setup and (f) immobilized packed bed photoreactor.

Ag/AgBr/TiO_2_ photocatalyst was synthesized using a modified protocol based on our earlier work.^25^ For immobilization, 3 mm solid soda lime #3000 glass beads (Walter Stern Inc.) were washed in 28–30%, NH_4_OH (VWR) and left soaking for 12 h followed by hot air oven drying at 70 °C. 100 dried beads were weighed (*W*_1_) and added to a 3 mM cetyltrimethylammonium bromide (CTAB, >99%, Aldrich) suspension and left washing for 1 h. Diamine silver ion solution, adjusted for 50 wt% AgBr solution, was added to the beaker containing CTAB bead suspension. A sol suspension of 0.5M TiO_2_ with diethanolamine (DEA) in a ratio of [DEA]/[TiO_2_] = 4 was added and stirred for 2 h. MilliQ water was added dropwise to maintain [H_2_O]/[TiO_2_] = 2 or until a clear sol was obtained. The sol was added to Ag-CTAB bead suspension. To promote rapid formation of Ag nanoparticles, photoreduction of the colloidal solution^41^ was initiated by exposing the beaker to 300W, ozone emitting Xe Lamp fitted with a 250 nm cut-off dichroic filter (Newport) for 10 min. The beaker with reduced Ag solution was finally left stirring for 12 h and calcined thereafter for 3 h (ramp rate of 5 °C min^−1^ until 500 °C). The dry weight of Ag/AgBr/TiO_2_ immobilized beads was recorded as *W*_2_ and catalyst loading was determined. Adhesion of the Ag/AgBr/TiO_2_ coating on the glass beads was studied using cross-cut test according to ASTM D3359 (ref. 42) Scotch Tape Test (3M 250 3710 Scotch tape). The weight of Ag/AgBr/TiO_2_ immobilized beads post photocatalytic treatment was recorded as *W*_3_ and presented in Table 2. Ammonia-washed uncoated glass bead is shown in Fig. 1(a). Fig. 1(b) and (c) show the scotch tape tested photocatalyst coated glass beads. Fig. 1(d) shows glass beads under grazing light illuminated microscope.

**Table 2:** Operating parameters of the photocatalytic reactor and catalyst properties in this work

Feed flowrate *F* (L h^−1^)	Bed porosity *∈*_b_	Residence time, *τ* (sec)	Catalyst loading, *C*_cat_ (g mL^−1^)	Catalyst surface area (m^2^ g^−1^)	Bead surface area (cm^2^)	Light intensity (*W*)	Photon flux (µmol s^−1^)	Weight of glass beads (g)		
								*W*_1_	*W*_2_	*W*_3_
68.1	0.30	0.3	0.61	26.97	1.13	1000	0.0376	2.982	3.863	3.247

### 2.2. Photocatalytic reactor setup and experiments

A bench scale system consisting of a 1 L holding tank and a recirculating packed bed photoreactor was developed as shown in Fig. 1(e). Glass beads immobilized with Ag/AgBr/TiO_2_ photocatalyst were packed in the 2.2 cm diameter and 20 cm long quartz tube (refractive index 1.46). Stainless steel wool was used as space packings to hold the beads stable during flow in the photoreactor. The beads filled an effective length of 15 cm covering the illumination spread of the lamp for maximum light delivery as shown in Fig. 1(f). A 1000 W, Xe arc lamp (*λ*_min_ = 260 nm, 5500-6000 K radiations) was used as the light source for top–down irradiation. A Teflon coated centrifugal pump (Cole-Parmer) circulated the wastewater. All piping and compression fittings were made in 316 stainless steel. Inlet and outlet pressures of the photoreactor were measured using pressure gauges P1 (Silicone filled vibration resistant, 0-300psi, McMasterCarr) and P2, respectively. The reactor was manually rotated at 2 rpm to maximize light exposure on glass beads.

Wastewater obtained from an industrial facility was used as the matrix for the spiked model compound to account for background effects. The concentration of dissolved oxygen in wastewater was measured using a Traceable 4320 Dissolved Oxygen meter (ITM Instruments Inc.) and pH was measured using Oakton pH 5+ meter. The wastewater matrix was spiked with 400 µM carbazole (CAB) chosen as the heterocyclic model compound. Experiments were carried out at 7.0–7.5 pH and room temperature (20–22 °C), with a stable dissolved oxygen of 2 ppm maintained throughout the reaction. The system was operated for 15 min. at the target flowrate in order to achieve steady-state conditions before sampling. A list of the investigated process conditions and photocatalyst properties is shown in Table 2. After the irradiation period, samples were collected from the holding tank for EPR spectroscopy.

Photocatalytic conversion of the model compound was evaluated in terms of apparent quantum efficiency:^43^1
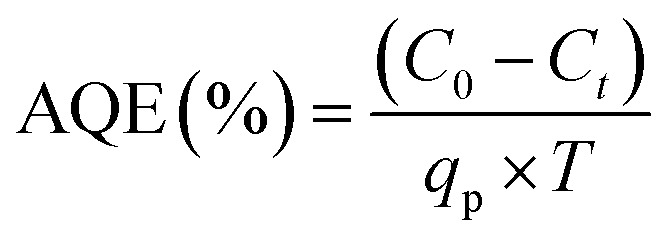
where, *C*_0_ is the initial and *C*_*t*_ are the initial and final concentrations of CAB determined from the fluorescence spectra of wastewater recorded on Tecan infinite 200 pro (emission *λ*:400 nm and excitation *λ*: 310 nm). *q*_p_ is the photon flux for the Xe-Arc lamp and was calculated as 0.0376 µmol s^−1^. *V* is the feed volume of wastewater circulated for *T* hour. All relevant calculations are shown in Table S2.

### 2.3. Scavenger tests and quenching studies

Dominant ROS driving the photocatalytic oxidation were identified through a scavenger test. A 400 µM solution of CAB was dissolved in Milli-Q water pre-charged with DMPO and illuminated with a catalyst loading of 0.5 g L^−1^. Potassium dichromate (K_2_Cr_2_O_7_) and ethylenediamine tetra acetic acid disodium salt (EDTA-2Na) were added to the spiked Milli-Q water to scavenge e^−^ and photogenerated h^+^, respectively. The resulting ROS formed spin-adducts with DMPO, which were subsequently detected *via* EPR spectroscopy.

The mechanism of ROS attack on CAB was elucidated through quenching studies. EDTA-2Na (EDTA), 2-propanol (IPA), *para*-benzoquinone (BQ), and AgNO_3_ were used as quenchers for h^+^,˙OH, O_2_˙^−^ and e^−^, respectively. The quenching effect was estimated by fluorometrically measuring the percentage change in CAB concentration between the start and end of the experiment.

### 2.4. Electron paramagnetic spectroscopy

**Fig. 2:**
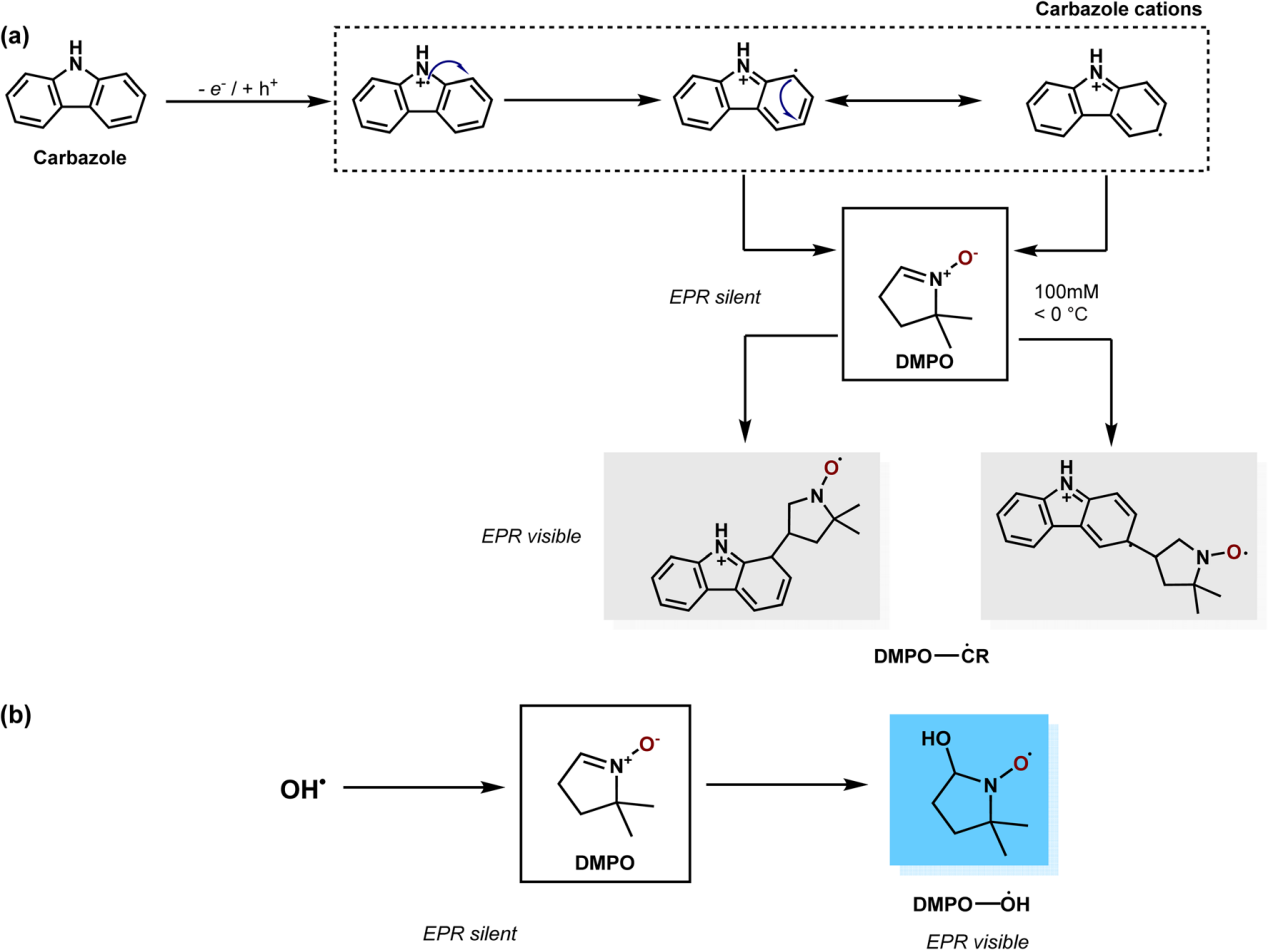
Radical trapping mechanism showing the formation of (a) carbon centered; DMPO-˙CR and (b) oxygen centered; DMPO-˙OH adducts. Both these DMPO adducts can be detected by EPR spectroscopy. Carbazole cation resonate among three different structures.

Free radicals generated during the photocatalytic oxidation of CAB were stabilized using 5,5-dimethyl-1-pyrroline *N*-oxide (DMPO) as a spin-trapping agent. DMPO forms stable adducts with free radicals, which exhibit hyperfine splitting when probed in an EPR spectrophotometer. Fig. 2 illustrates the mechanism of DMPO-˙OH and DMPO-˙CR adduct formation *via* trapping of photogenerated hydroxyl and carbazole cation radicals by DMPO. The trapped free radicals were identified based on their characteristic peak shape which appears in the EPR spectra. By double integrating the peak area of the free radical-DMPO adducts in the spectra, radical concentration relative to a standard calibration was determined. The list of peak intensity ratios of DMPO adducts characterized in this study are presented in Table S3. The relation between DMPO-adduct concentration ([DMPO-adduct]) and peak area in EPR spectra 
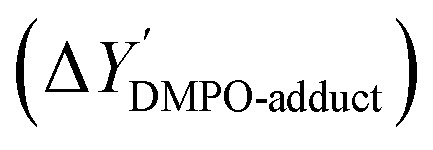
 is given by the following relation:^44^2
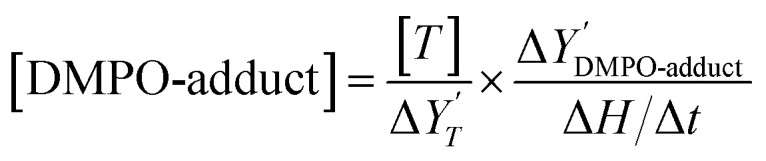


TEMPOL was used for calibration and the relation between tempol concentration [*T*] and their peak area 
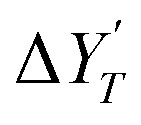
 was obtained from the calibration plot shown in Fig. S4. Δ*H*/Δ*t* is the conversion factor between the calibration and actual EPR spectra. The conversion factor between the two derivatives is time rate of field sweep presented in the supplementary material.

All EPR spectra of radical-adduct signals were recorded on a Bruker ECS-EMX X-band EPR spectrometer equipped with an ER4119HS cavity. Typical operating parameters were as follows: microwave frequency 9.34 GHz, microwave power 2.15 mW, modulation amplitude 4 G, sweep width 100 G, attenuation/gain 20/30 dB. *g*-Values were determined by calibration with DPPH (2,2-diphenyl-1-picrylhydrazyl), a stable organic radical in solid form. Spectra were acquired in a field sweep mode using the following parameters: Ten 60 s scans were averaged together at 180° phase offset for a total acquisition time of 600 s. The quality factor for the acquired spectra ranged between 1700–1900.

Curve fitting on the EPR spectra was done using OriginPro 2024b to determine the transient concentration of ˙OH and ˙CR radicals. 3–10% end point weighted baseline correction was first applied to all EPR spectra. After baseline subtraction, ˙OH, and ˙CR peaks were identified based on simulated data and fitted with Fraser–Suzuki asymmetric function represented by the following formula for peak deconvolution:^45^3
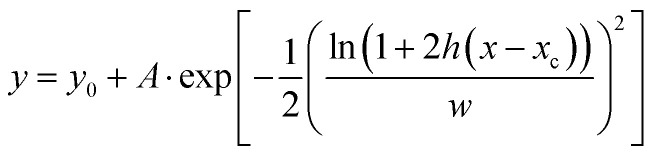
where, *y*_0_ is the baseline offset, *A* is the amplitude, *h* is the asymmetry factor, *x*_c_ is the peak center, and *w* is the peak width.

### 2.5. Mechanistically derived spectro-kinetic modelling

Mechanistic spectro-kinetic model assumes a Micro Steady State (MSS) approximation between the photo generated charge carriers; h^+^ and e^−^, free radicals; ˙OH and ˙CR, and the model compound; X. The proposed mechanism of photocatalytic oxidation is outlined in Table 3.

**Table 3:** Proposed photocatalytic oxidation mechanism

Reaction step	Rate constant	
Activation step:		
Photocatalyst + *hν* → h^+^ + e^−^	*k*_o_	(v)
Electron capture step:		
O_2_ + 3e^−^ + 2H^+^ → ˙OH + OH^−^	*k*_1_	(vi)
Recombination step:		
e^−^ + h^+^ → heat	*k*_2_	(vii)
Oxidation step:		
X + h^+^ → ˙CR + products	*k*_3_	(viii)
X + ˙OH → ˙CR + products	*k*_4_	(ix)
Termination step:		
˙OH + ˙CR → products	*k*_5_	(x)

The model compound (X) can undergo oxidation *via* two main pathways: (1) direct oxidation by photo generated holes formed during irradiation, or (2) e^−^ transfer mediated oxidation by hydroxyl radicals. Both pathways can occur simultaneously and compete in the photocatalytic oxidation process. Pathways for hydroxyl radical formation is usually dictated by thermodynamic favourability. A hole-mediated water splitting, or a 3-step electron mediated oxygen reduction reaction^12,46^ could equally be favored to produce hydroxyl radicals. Oxidation of CAB used as the model compound in this study, forms carbazole cation radicals as intermediates^47^ (represented as ˙CR, CR/˙CR = −0.80 V *vs.* NHE which subsequently mineralizes into carbon dioxide and organics (products). To account for overall species balance, our model assumes that the termination reaction (*x*) and recombination reaction (vii) compete with the main oxidation reactions (viii) and (ix).

Based on the oxidation of model compound by h^+^and ˙OH, the following rate expression is derived for ˙OH:4
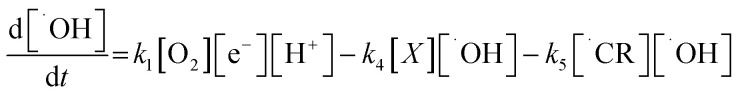


Note that electron capture reaction, reaction (viii), is sum of several elementary reaction. However, in the above expression, a simplified reaction rate expression is used. For ˙CR:5
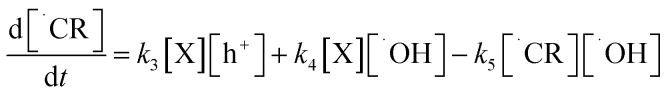


Similarly, the rate expression for charge carriers can be derived:6
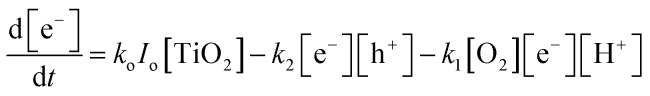
and:7
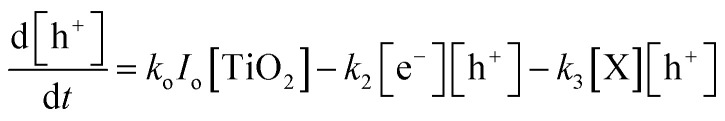
where *I*_o_ is the light intensity and *k*_o_ is the pseudo-first-order rate constant for photocatalyst activation^48^ estimated by equations presented in S5.

Considering the equilibrium, the superficial oxidation reaction rate of *X* through both oxidation pathways expressed in reactions (x) and (xi) is given as:8
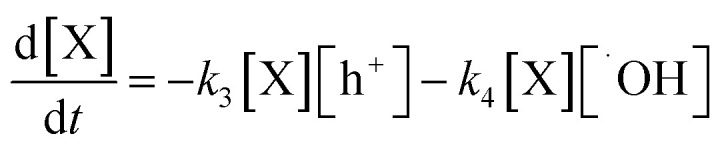
In order to evaluate the degradation rate of compound X, the rate of disappearance by direct hole attack, *k*_3_, and hydroxyl radical oxidation, *k*_4_ must be determined. Upon irradiation, shallowly trapped holes, attain a thermally activated equilibrium with free holes exhibiting a very high oxidation potential, hence hole concentration remains constant:^1,49^9
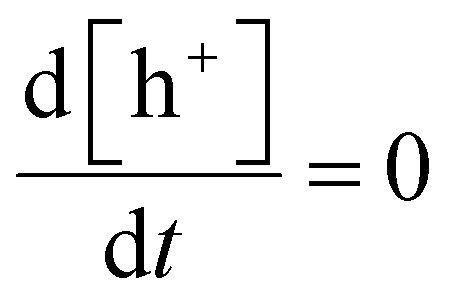


From eqn (8) and (11), the rate constant for primary oxidation can then be expressed as;10
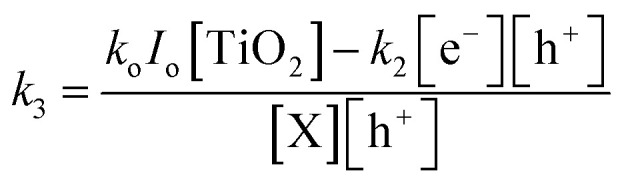


And secondary oxidation as;11
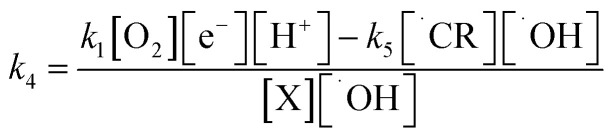


Thus, the final superficial oxidation reaction rate of contaminant X is expressed as:12
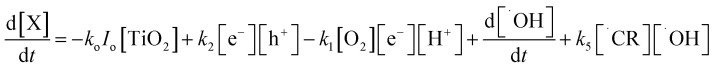


The above equation was solved using a built-in ordinary differential equation solver (ode45) in MATLAB R2024b with relative tolerance of 1 × 10^−3^ and absolute tolerance of 1 × 10^−6^. Kinetic parameters and other constants were obtained from the literature, as outlined in Table 4.

**Table 4:** Values of constant and assumptions used in kinetic modeling

Constants	Value	Assumption	Reference
*k*_0_	6 m^2^ g^−1^	Photocatalyst particles are spherical in shape. Rate of activation of e^−^/h^+^ follows a pseudo first order rate constant. Further details are presented in S5	48
*I*_o_	4.96 × 10^−11^ µm^−2^ mol^−1^ h^−1^	Spectral radiance of ozone free Xe-arc lamp at 50 cm distance from reactor	This work
*k*_1_	183.6 h^−1^	Rate of e^−^ charge transfer was adopted from our previous work	25
*k*_2_	97.2 h^−1^	Rate of recombination was adopted from our previous work	25
*k*_5_	36 × 10^6^ µM^−1^ h^−1^	CAB cations unselectively quench ˙OH at diffusion-controlled limits. A representative second order quenching rate constant was assumed. Further details are presented in S1	50
[h^+^]	0.3 µM	[h^+^] contributing to the formation of ˙CR radicals in the absence of e^−^ during quenching studies was obtained from the EPR spectra	This work
[O_2_]	31.2 µM	Dissolved oxygen measured in the wastewater sample	This work
[H^+^]	0.03 µM	Determined from the pH of the wastewater sample	This work

## 3. Results and discussions

### 3.1. Band positions of Ag/AgBr/TiO_2_ photocatalyst

**Fig. 3:**
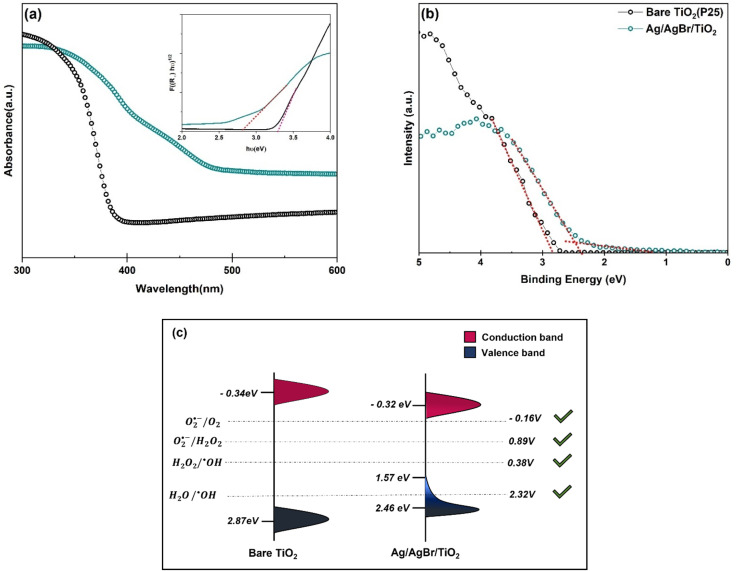
The (a) UV-DRS spectrum and (b) VB XPS of bare and as prepared Ag/AgBr/TiO2 catalyst along with the (c) energy diagram and band edge reduction potential *vs.* NHE for ROS generation.

Fig. 3(a) shows the UV-vis DRS spectra of the Ag/AgBr/TiO_2_ photocatalyst. The bare TiO_2_ exhibited absorption edges at about 380 nm. Comparatively, the absorption edge of Ag/AgBr/TiO_2_ heterojunction was expanded to 500 nm, indicating that the addition of AgBr played a significant effect on the optical property in the visible light region. Inset in Fig. 3(a) shows the *Tauc* plot which is obtained by the conversion of reflectance spectra using Kubelka–Munk method.^51^ For indirect transitions the optical band gaps of pure TiO_2_ and Ag/AgBr/TiO_2_ were reported as 3.21 eV and 2.78 eV, respectively. The addition of silver halide groups has been reported to reduce the bandgap of TiO_2_ photocatalyst and increase their visible light activity. Different phenomenon such as plasmonic resonance, band bending and Urbach band tailings have been reported to influence the optical activity. However, the density of states (DOS) band positions influences the redox abilities and charge transfer mechanism of the photocatalysts strongly than all other phenomena. The XPS survey spectra of the reduced Ag/AgBr/TiO_2_ photocatalyst are presented in Fig. S6 together with Table S7, confirming the atomic percentages of Ag, Br, Ti and O. The valence band XPS spectrum shown in Fig. 3(b) presents valence band edge of bare TiO_2_ at maximum energy of approximately 2.78 eV, which is in good agreement with values reported in the literature.^52^ Since the optical bandgap of TiO_2_ is 3.12 eV, the conduction band minimum would occur at −0.34 eV. On the other hand, the valence band edge of Ag/AgBr/TiO_2_ was estimated to be at 2.44 eV but followed by a band tail much further towards ∼1.07 eV. Such band tailing has been reported in other TiO_2_ based heterocomposites.^53^ Together with optical measurement results indicating a narrowed band gap, the conduction band (CB) minimum of the reduced Ag/AgBr/TiO_2_ is estimated to be at −0.32 eV using the method reported in previous reports^54,55^ and calculated in Table S8. A schematic illustration of the DOS of bare and Ag/AgBr/TiO_2_ is shown in Fig. 3(c).

Based on the band positions, the photogenerated e^−^ and h^+^ formed on the surface of Ag/AgBr/TiO_2_ photocatalyst can produce different ROS which can oxidize heterocyclic CAB compound. Superoxide anion (O_2_˙^−^) are formed by the reduction of dissolved oxygen by photogenerated e^−^ while hydroxyl radical (˙OH) are produced either from the splitting of adsorbed water molecules by h^+^ (reaction (i)) or by a 3 e^−^ step reduction of dissolved oxygen (reaction (i)–(iii)).^33,56^ Since the conduction band potential of Ag/AgBr/TiO_2_ (*E*^0^_CB_ = −0.32 eV) is more negative than the standard reduction potential of O_2_/O_2_˙^−^ (–0.16 V *vs.* NHE^57^) the conduction band electron can reduce oxygen to produce O_2_˙^−^ radicals. Further reduction of O_2_˙^−^ to ˙OH *via* H_2_O_2_ is also possible. The valence band potential of Ag/AgBr/TiO_2_ (2.46 eV) is much higher than the standard redox potential of H_2_O/˙OH (+ 2.34 V *vs.* NHE) and ^−^OH/˙OH (+1.99 eV *vs.* NHE),^58^ thus formation of ˙OH *via* water splitting by h^+^is another possibility of ROS formation pathway. Among the possible ROS formation, production of ˙OH *via* H_2_O_2_ in a 3 e^−^ step reduction of dissolved oxygen is less kinetically favoured over h^+^ mediated water splitting.^59^ The initial reduction of Ag^+^/Ag^0^ occurring at −0.8 V provide plasmonic resonance effect.

### 3.2. Mechanistic interpretation of oxidative pathways

**Fig. 4:**
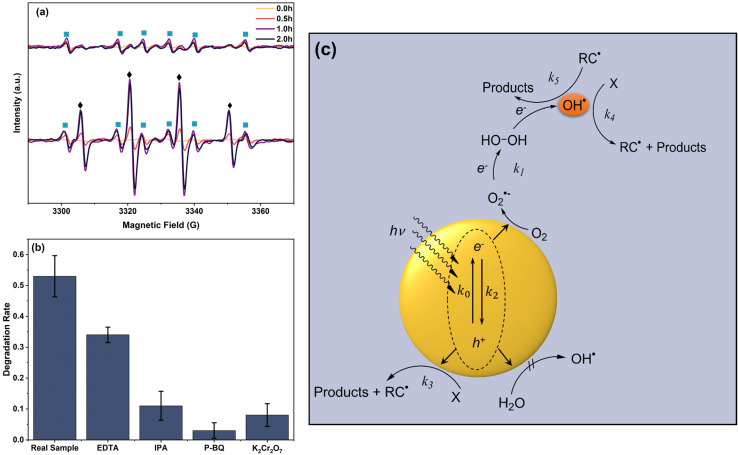
(a) EPR spectra of e^−^ scavenged (top) and h^+^ scavenged (bottom) Ag/AgBr/TiO_2_ photocatalyst, (b) scavenger test for CAB oxidation, and (c) proposed reaction schematic of ROS generation pathways under visible-light irradiation in the Ag/AgBr/TiO_2_ system with their hypothesized kinetic rate constants.

The temporal EPR spectra obtained from the scavenger tests as shown in Fig. 4(a) indicated that upon adding EDTA-2Na, the peaks of CAB cation radical (DMPO-˙CR) and hydroxyl radical (DMPO-˙OH) adducts appeared with sustained peak intensity at 1.0 h. Intriguingly, upon dosing K_2_Cr_2_O_7_ in the photocatalyst reaction system, the EPR spectra exhibited a pronounced peak of DMPO-˙OH radical along with DMPO-˙CR. The peak intensity of CAB cation radicals stayed constant while the peaks of hydroxyl radicals showed maximum sustained intensity starting at 1.0 h and continued till the end of reaction period. Two key factors were identified from this observation. Firstly, hydroxyl radical which was the dominant ROS and was produced by the action of CB e^−^ and not h^+^. In particular, when ˙OH reached a quasi-steady state at 1.0 h, the peak areas of ˙CR showed broadening, signifying enhanced oxidation intensity than in the absence of ˙OH. Secondly, in the absence of ˙OH, CAB still undergoes decomposition, producing a steady-state concentration of CAB cation radicals as intermediate. This indicates that a secondary reaction mechanism also promotes the photocatalytic decomposition of CAB in Ag/AgBr/TiO_2_ system. The true oxidation potential of photogenerated h^+^ is difficult to experimentally compute. However, studies have shown that high-energy photogenerated holes exhibit scalable oxidation applicability, such as the degradation of organic pollutants in water under natural sunlight.^58^ Reaction of h^+^ driving the oxidation of phenol and subsequently generating phenoxyl radical have been reported to occur at *E*_0_ of 1.76 V.^60^ Moreover, due to their relatively short lifespan, h^+^ can potentially drive oxidation in the presence of natural free radical scavengers such as humics.^61^

In the absence of any quenchers, 54% of spiked CAB in wastewater underwent photocatalytic oxidation as shown in Fig. 4(b). Upon adding, EDTA, only 33% of CAB was oxidized which further decreased to 10% in the presence of IPA. The addition of K_2_Cr_2_O_7_ had a similar effect. When O_2_˙^−^ was trapped by BQ, the photocatalytic oxidation was almost completely inhibited with less than 1% CAB undergoing oxidation. The weaker inhibiting effect of EDTA supported h^+^ as minor contributing species to CAB oxidation. In contrast, quenching of e^−^, ˙OH,and O_2_˙^−^ significantly reduced the photocatalytic oxidation O_2_˙^−^ quenching leading to maximum inhibition. The superoxide anion is most likely the key intermediate in the formation of ˙OH, hence its elimination completely suppressed the oxidation of CAB. O_2_˙^−^ can oxidize CAB directly or undergo further reduction to form ˙OH.^62^ This was witnessed in the presence of IPA, when ˙OH was scavenged but the oxidation of CAB continued albeit at a lower rate. O_2_˙^−^ oxidized CAB at a lower rate due to its lower oxidation potential than ˙OH. Similarly, when the e^−^ was scavenged by K_2_Cr_2_O_7_, the production of ˙OH, *via* O_2_˙^−^ and H_2_O_2_ as an intermediate was suppressed. Simultaneously, the e^−^/h^+^ recombination reduced and the contact efficiency between h^+^ and adsorbed species increased thereby promoting the oxidation of CAB. Fig. 4(c) presents the reaction schematic along with the ROS for the oxidation pathway of CAB. The direct oxidation pathway implies that CAB molecules adsorbed at the surface of the photocatalyst are oxidized by h^+^ spontaneously, rapidly achieving steady-state, as seen in Fig. 3(a). The indirect oxidation pathway of ˙OH by O_2_˙^−^ although predominant, could only achieve quasi-steady state conditions. Reports also suggest that surface generated ˙OH are more beneficial for adsorbed reactants.^63^ ˙OH formed *via* O_2_˙^−^ pathway is more likely to exist in freely dissolved state and may not be as effective in photocatalytic oxidation as a h^+^ mediated surface generated ˙OH.^63^

### 3.3. Detection and evolution of free radicals

**Fig. 5:**
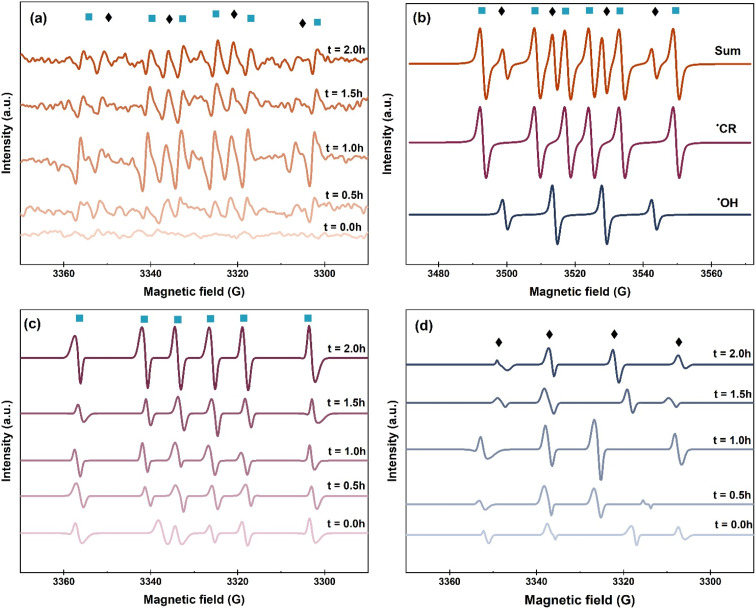
EPR spectra of coking wastewater evolving over reaction time of 2 h. (a) Measured EPR spectra, (b) simulated EPR spectra, (c) deconvoluted ˙CR peaks, and (d) deconvoluted ˙OH peaks.

Fig. 5(a) illustrates the signals of DMPO-radical adducts detected in the EPR spectra over the entire reaction time. Peaks of DMPO-˙OH were recognized by the classical quartet peaks of 1 : 2:2 : 1 intensity ratio^64^ and DMPO-˙CR peaks were identified by sextet peaks of 1 : 1:1 : 1:1 : 1 intensity ratio.^65^ The sum of simulated DMPO-˙OH and DMPO-˙CR peaks shown in Fig. 5(b) matches the observed EPR signals further confirming the presence of hydroxyl and CAB cation radicals as the dominant free radical species in the wastewater media.

The deconvoluted peaks of ˙CR and ˙OH radicals are presented in Fig. 5(c) and (d), respectively. Temporal evolution of ˙CR peaks started with a baseline signal and peak intensities evolved strongly over the reaction time. In case of ˙OH radicals, the peak intensities were strongest from baseline measurements at 1.0 h reaction time interval and subsequently decreased. The persistent increase of ˙CR radical can be ascribed to their formation by the photocatalytic oxidation of CAB. ˙OH radical was the primary ROS which photo-oxidized CAB into ˙CR intermediate. The decline in the peak intensity of ˙OH was possibly due to its high oxidation potential (2.8 V) which makes it non-selective.^66^ Thus ˙OH was likely consumed in the reaction by ˙CR intermediate and other substrate present in the wastewater matrix. As ˙OH continued to decline, oxidation of CAB shifted towards the secondary pathway mediated by direct h^+^ oxidation.

### 3.4. Validation of spectro-kinetic model

**Fig. 6:**
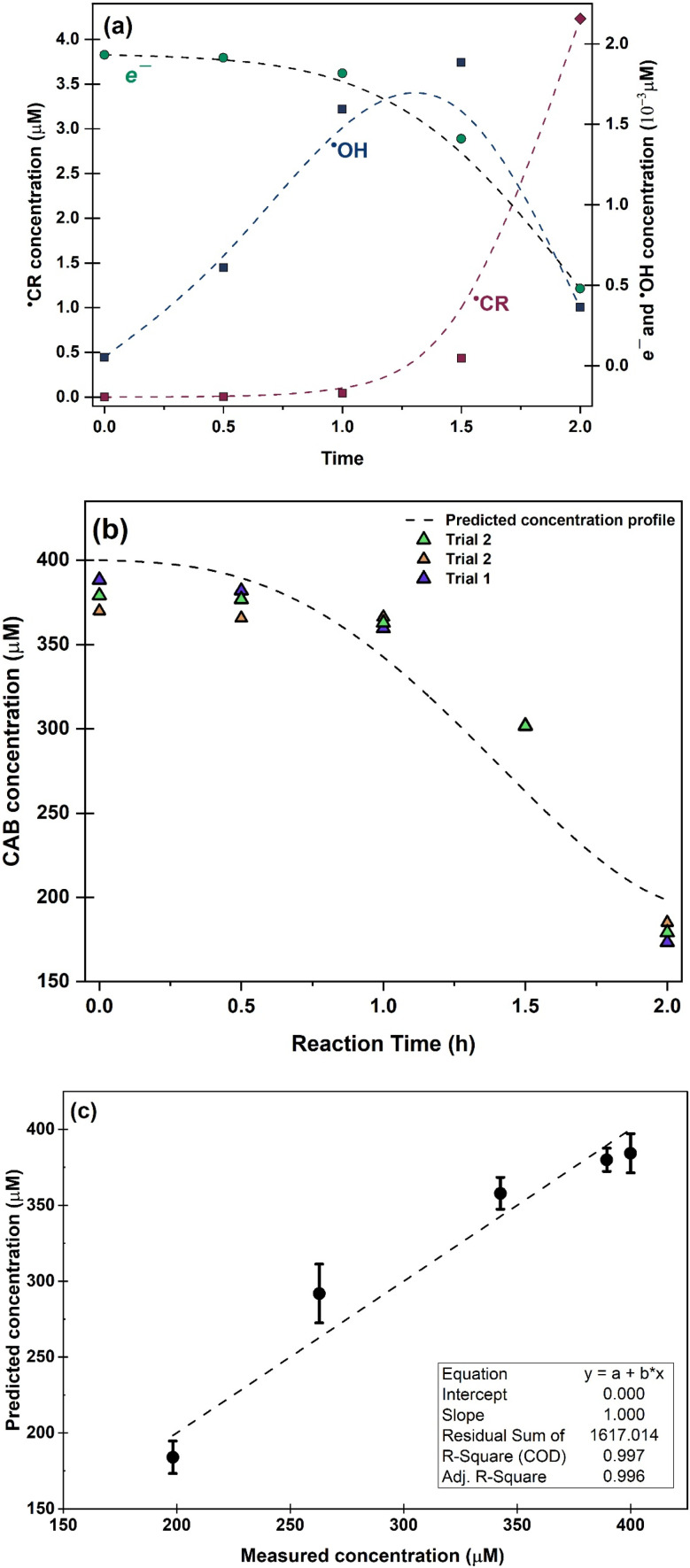
(a) Evolution of photogenerated e^−^ charge carries and free radical species taking part in the photocatalytic oxidation, (b) kinetic plot showing experimental and predicted CAB concentration during the photocatalytic reaction (c) Parity plot of predicted and experimental concentration of CAB during the 2.0 h reaction period.

Temporal concentration of ˙OH and ˙CR free radicals was determined by double integrating the deconvoluted peak areas of DMPO-˙OH and DMPO-˙CR in the continuous wave EPR (Fig. 5(c) and (d)). The peak fits obtained during deconvolution are shown in Fig. S9. Peak areas of DMPO-˙OH and DMPO-˙CR was converted to radical concentration against nitroxide 4-hydroxy tempo (Tempol) as the calibration standard.^67^ Since the photogenerated e^−^ attained a quasi-steady state during the scavenger test leading to the evolution of DMPO-˙OH peaks in Fig. 4(a), the concentration of e^−^ was obtained by double integrating the ˙OH peaks and equating the steady-state ˙OH concentration to the concentration of e^−^. Fig. 6(a) shows the changing concentration of ˙OH, ˙CR and e^−^ over the 2.0 h reaction time. The dashed line is the continuous fit function obtained by fitting the temporal concentration to an empirical model by least squares regression method. Table 5 outlines the fitted equations of ˙OH, ˙CR and e^−^ kinetics and the goodness of fit obtained. The fitted functions were used to solve eqn (12) and predict the concentration of CAB at different time intervals.

**Table 5:** Kinetic fitting functions for each ROS and charge carrier with their goodness of fit

Species	Function	Kinetic function	*R*_2_ (COD)
CB electrons (e^−^)	Boltzmann	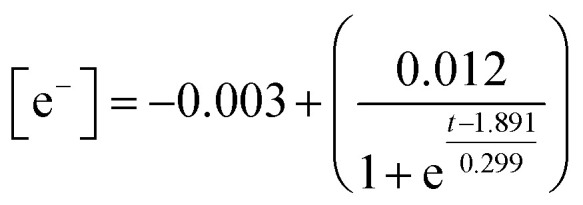	0.99
CAB cation radicals (˙CR)	Exponential growth	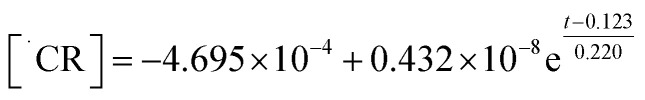	0.99
Hydroxyl radicals (˙OH)	Cubic spline	[˙OH] = 2.659 × 10^−4^ − 2.766 × 10^−4^*t* + 0.015*t*^2^− 0.007*t*^3^	0.94

From Fig. 6(a) and Table 5 it can be concluded that the concentration of ˙OH decayed after 1.5 h following a continuous rise in concentration initially. Such behaviour of ROS has been reported previously^66,68,69^ and has been found to be dependant primarily on the mechanism of ROS formation. In particular, dissolved oxygen has been reported as a limiting reagent which leads to a decay in the ˙OH generation after the initial oxygen is consumed by the photogenerated e^−^. Occurrence of non-targeted secondary reactions leading to the consumption of excess ˙OH in the system cannot be ruled out^64^ especially in industrial wastewater which contains high levels of organic carbon. In this study, both these phenomena could be contributing to the consumption of ˙OH.

Photogenerated CB e^−^ also showed a decay after 1.0 h. For such a decline in the steady-state concentration of e^−^, there are two possible factors: (1) irreversible change in the oxidation state from Ag^+^ to Ag^0^ in the catalyst, or (2) inhibition of e^−^charge transfer to ˙OH radical formation. Ag/AgBr/TiO_2_ has been reported to display localized surface plasmonic resonance phenomenon upon illumination. This has been confirmed in our earlier works^25^ by charge transfer analysis. As the reaction progresses, some amount of AgBr irreversibly degrades to form Ag nano-islands on the photocatalyst surface.^13^ Metallic silver could act as a sink for hot-electrons and reduce electron injection to the conduction band of TiO_2_, thereby decreasing the net negative charge transfer during illumination. On the other hand, since we estimated the concentration of e^−^ by assuming the steady state formation of ˙OH radicals which itself declined, thus the kinetics of photogenerated e^−^ might be following in tandem the kinetics of ˙OH radical formation. However, the concentration of e^−^ was determined in pure MilliQ water without any interference from wastewater matrix. Hence, it is likely that factor (1) might be the correct explanation for the observed e^−^ kinetics. ˙CR radicals, continued an exponential rise until the end of the reaction period. This indicates that although the ˙OH radical declined after 1.0 h, photocatalysis of CAB continued to progress, possibly through secondary oxidation of CAB by direct h^+^ attack. Additionally, as the ˙OH concentration declined, the quenching reaction of ˙OH consuming ˙CR radicals were interrupted and the concentration of ˙CR increased exponentially.

Fig. 6(b) presents the comparison of CAB concentration obtained experimentally and the predicted CAB concentration from the developed kinetic model. The comparison results showed that the estimated values by the spectro-kinetic model were consistent with experimental data, indicating that the developed model was able to estimate the photocatalytic oxidation of CAB in the wastewater matrix. Fig. 6(c) illustrates the parity plot of experimental and predicted CAB concentration during the reaction time interval. The experimental values were within close range of the predicted values. In conclusion, the kinetic model can be used to describe the photocatalytic conversion of CAB following the free radical mediated oxidation pathways. The AQE of the validation data was determined to be 75%.

### 3.5. Effect of kinetic rate constants

**Fig. 7:**
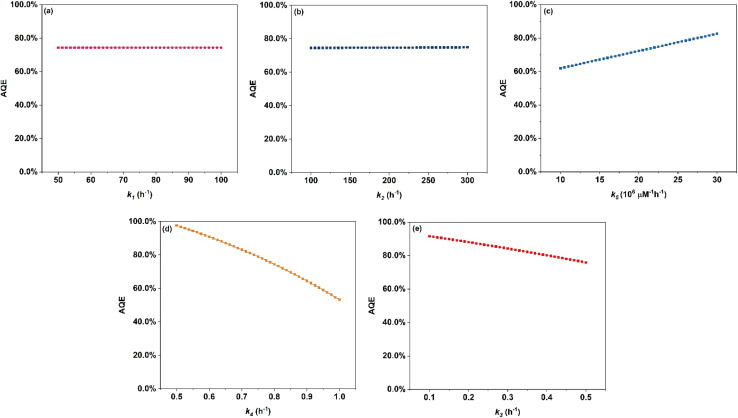
Parametric study of rate constants (a) *k*_1_, (b) *k*_2_, and (c) *k*_5_. Effect on AQE by varying kinetic constants for primary oxidation (d) *k*_4_ and secondary oxidation (e) *k*_3_ of CAB.

The apparent quantum efficiency for Ag/AgBr/TiO_2_ photocatalysts for continuous flow packed bed reactor in this study was around 70%. Other studies have reported AQE in the range of 30–90% as shown in Table S10. Depending on the photocatalytic system and the application of oxidation potential towards targeted application leads to varying AQE in literature. Fig. 7(a)–(c) illustrates the effect of varying *k*_1_, *k*_2_, and *k*_5_ rate constants, on the AQE of photocatalytic oxidation. Among these, *k*_5_ which represents the quenching of ˙OH radicals during the termination reaction (xii) had maximum impact on the AQE of photocatalytic oxidation. In contrast, an increase or decrease in the kinetics of charge transfer (*k*_1_) and recombination (*k*_2_) rate showed no influence on the AQE of photocatalytic oxidation. This could indicate that a higher presence of photogenerated charge carriers (e^−^/h^+^) may not directly coincide with a higher AQE. Additionally, the benefit of high oxidation potential of ˙OH radicals can be offset by their shorter lifetime in the presence of excessive quenchers. Fig. 7(d) and (e) illustrates the predicted change in the AQE of photocatalytic oxidation by varying the rate constant of hydroxyl radical (*k*_4_) and direct hole attack (*k*_3_) oxidation pathways. 40% change in AQE was predicted by varying *k*_4_ whereas a change in *k*_3_ was predicted to cause 20% change in AQE. This indicates that oxidation *via* ˙OH (reaction (ix)) is likely the faster route, compared to direct hole attack (reaction (viii)). Hence ˙OH mediated oxidation is identified as the primary pathway. Direct hole attack plays a secondary role in the photocatalytic oxidation of CAB. Although the formation of ˙OH follows a more complex and thermodynamically less favorable 3 e^−^ step transfer, it is kinetically favoured over direct h^+^ attack of CAB.

## 4. Conclusion

In this study, a novel approach for spectro-kinetic model was developed and validated using experimental data from a continuous-flow packed bed reactor setup featuring immobilized Ag/AgBr/TiO_2_ photocatalyst on glass beads. The experiments were carried out at room temperature with 400 µM spiked model compound CAB in wastewater media. ROS species and free radical intermediates formed under visible light illumination were characterized and quantitated by EPR spectroscopy under micro steady state conditions.

Based on the band position of the Ag/AgBr/TiO_2_ photocatalyst, three possible ROS formation mechanism were hypothesized; (1) formation of O_2_˙^−^ radical by SET reduction of dissolved oxygen by conduction band e^−^. (2) Formation of ˙OH radical from O_2_˙^−^*via* peroxide (H_2_O_2_) reduction. (3) Formation of ˙OH radical *via* water splitting by valence band h^+^. Scavenger tests conducted using radical quenchers identified ˙OH radical as the predominant ROS with O_2_˙^−^ and H_2_O_2_ acted as intermediates. This suggests that ˙OH radical formation progressed *via* kinetically less favorable 3 e^−^ oxygen reduction reaction. Under the investigated conditions, 54% of CAB was oxidized, achieving an apparent quantum efficiency (AQE) of 75%. The photocatalytic oxidation of CAB followed multiple parallel-sequential pathways, including ˙OH, ˙CR free radicals and photogenerated e^−^ as charge carriers.

Parametric study of kinetic rate constants confirmed that ˙OH radical mediated oxidation was the primary reaction pathway, followed by a secondary contribution from direct h^+^ attack on surface adsorbed CAB. Quenching of ˙OH radical by intermediate ˙CR species showed maximum impact on the AQE of CAB oxidation establishing it as the rate determining step. Thus, the competing reactions by intermediates has a high rate and effect in photocatalytic oxidation of CAB indicating that the removal of intermediates is an important step in designing efficient and selective photocatalytic systems. The developed spectro-kinetic model showed good agreement with experimental data achieving an *R*^2^ of 0.99. These findings highlight the potential of immobilized photocatalytic systems for continuous flow oxidation processes.Future works should focus on integrating mechanistic insights related to light intensity and behavior of photogenerated charge carriers using techniques such as *in situ* photoluminescence spectroscopy and Raman analysis into the spectro-kinetic modeling framework.

## Conflicts of interest

The authors declare no conflicts of interest.

## Supplementary Material

RA-016-D5RA09830K-s001

## Data Availability

The data supporting this article have been included as part of the supplementary information (SI). The code for MATLAB can be found at https://github.com/Rohit994/MATLAB with https://doi.org/10.5281/zenodo.17956067. The version of the code employed for this study is version V1.1.1. Supplementary information is available. See DOI: https://doi.org/10.1039/d5ra09830k.
