# Supplementary material for: Spectro-kinetic modelling of photocatalytic oxidation of heterocyclic compounds in a continuous-flow packed bed reactor
Source: RSC Adv. 2026 Apr 17;16(22):20282–94. doi: 10.1039/d5ra09830k (PMC13089631; doi:10.1039/d5ra09830k)
Supplement: RA-016-D5RA09830K-s001 [file RA-016-D5RA09830K-s001.pdf]

Supplementary Information (SI) for RSC Advances.
This journal is © The Royal Society of Chemistry 2026

Supplementary Information

Spectro-Kinetic Modelling of Photocatalytic Oxidation of Heterocyclic Compounds in a Continuous-Flow Packed Bed Reactor

Rohit Pal[a], *Ramin Farnood* [a, *]

[a] Department of Chemical Engineering and Applied Chemistry, 200 College Street, Toronto, ON M5S 3E5, Canada.

S1. Determination of rate constant k_5 for ${}^{\bullet}OH$ scavenging by ${}^{\bullet}CR$

A general scavenging rate constant for ${}^{\bullet}OH$ by CAB cation (${}^{\bullet}CR$) in aqueous solution is not known. However, reaction rates for similar heterocyclics cations as reported in literature[1] is listed below:

1. Pyrrolidinium ion

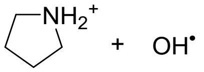

$5 \times 10^9\ M^{-1}s^{-1}$

2. Imidazolium ion

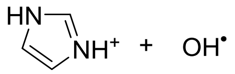

$5.5 \times 10^9\ M^{-1}s^{-1}$

Dissolved organics in effluents from wastewater treatment plant are known to scavenge ${}^{\bullet}OH$ radicals. The rate constant for dissolved organics quenching ${}^{\bullet}OH$ radicals has been proposed to be in the range of (0.2 -1.4) $\times\ 10^9\ M^{-1}s^{-1}$ [2–4]. Table S2 lists some of the common aromatic organic compounds found in coking wastewater and their ${}^{\bullet}OH$ scavenging rate constants from literature[1,5].

Aniline	$1.4 \times 10^{10}\ M^{-1}s^{-1}$
Benzene	$7.8 \times 10^{9}\ M^{-1}s^{-1}$
Ethanol	$1.9 \times 10^{9}\ M^{-1}s^{-1}$
Cresol	$1.2 \times 10^{10}\ M^{-1}s^{-1}$
Cyanide	$0.8 \times 10^{10}\ M^{-1}s^{-1}$
Phenol	$1.4 \times 10^{10}\ M^{-1}s^{-1}$
Thiocyanate ion	$1.1 \times 10^{10}\ M^{-1}s^{-1}$

Based on the above observations and structure property relationship with other heterocyclic ions, we assume a value of **1 × 10^{10} M^{-1}s^{-1}** as the rate constant for the $^{\bullet}OH$ scavenging by $^{\bullet}CR$. On a µM basis, $\boldsymbol{k_5}$ is calculated as **36 × 10^{6} µM^{-1} h^{-1}**.

S2: **Photon flux determination from discrete spectral irradiance**

The discrete spectral irradiance was obtain from Newport[6]. Photon flux can be calculated as:

$$q_p = \frac{I \times \lambda \times 5.03 \times 10^{15}}{6.02 \times 10^{17}}$$

Wavelength, λ (nm)	Intensity, I (mW/m²/nm)	Photon Flux, q_p (µmol/m².s)
250.74	17.45	0.04
299.82	119.97	0.30
350.55	162.79	0.48
400.74	203.85	0.68
449.26	200.60	0.75
499.45	251.19	1.05
550.74	217.37	1.00
600.37	213.91	1.07

The photon flux over the irradiant photoreactor area was determined to be 0.0376 µmol/s.

S3: **Peak intensity ratios and *g*-factor of ROS**

EPR spectrum	Peak intensity ratio	*g*-factor	Reference
DMPO-OH	1:2:2:1	2.0023	[7]
DMPO-OOH	1:1:1:1:1:1	2.004	[8]
DMPO-OCR	1:1:1:1:1:1	2.0027	[9]

S4: **Calibration plot of TEMPOL**

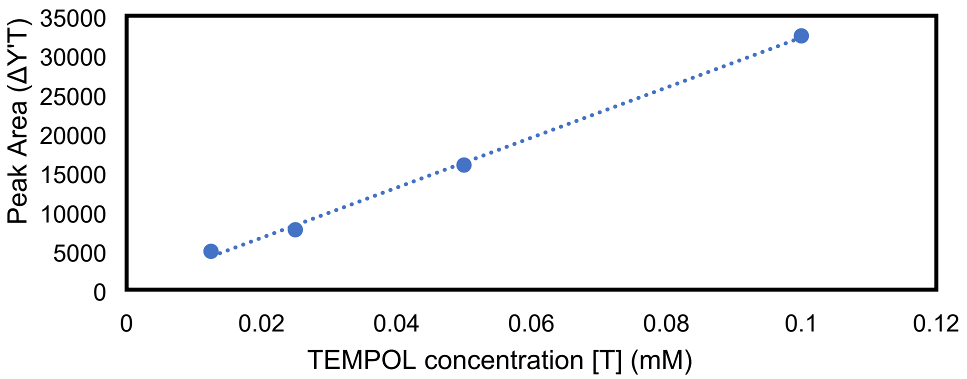

$\Delta H/\Delta t = 100/60 = 2.$

S5: Rate constant k_o for photocatalyst activation:

The value of k_o was estimated using the following equation by assuming photocatalyst particles are spherical in shape:

$$k_o = \frac{1}{4}\Phi\, Q_{abs}\, S_{BET}$$

Where Φ is the quantum efficiency, Q_{abs} is the absorption efficiency, and S_{BET} is the BET surface area of the catalyst. Using experimental data from our earlier study[10], k_o was estimated as ~6 m^2,g^{-1}.

S6: XPS survey spectra of reduced Ag/AgBr/TiO_2:

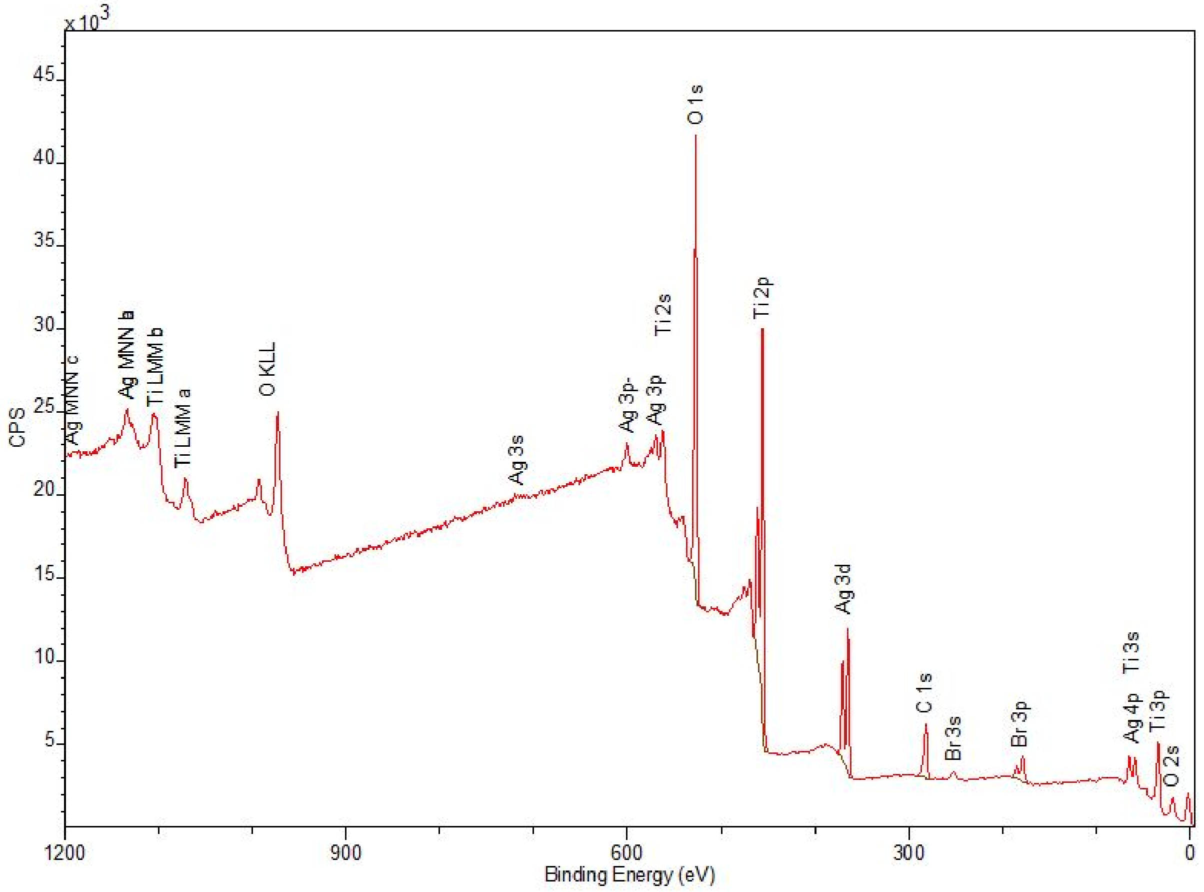

S7: Atomic percentages of Ti, O, Ag, Br calculated from XPS

Element	Relative Atomic %
Ti 2p	22.7
O 1s	49.7
Ag 3d	3.3
Br 3p	3.2

S8: **Band edge calculation:**

The position of the valence band, VB and the conduction band, CB can be determined, if the values of both the ionization energy and electron affinity are known. Conduction band potential is calculated as:

$$E_{CB}^{0} = E_{VB}^{0} - E_{g}$$

Where, E_{VB}^{0} is the valence band potential of photocatalyst[11] determined by VB XPS.

**Photocatalyst**	**Optical bandgap**	**CB potential**	**VB potential**
TiO_2	3.21	-0.34	2.87
$Ag/AgBr/TiO_2$	2.78	-0.32	2.46

S9: Peak Deconvolution

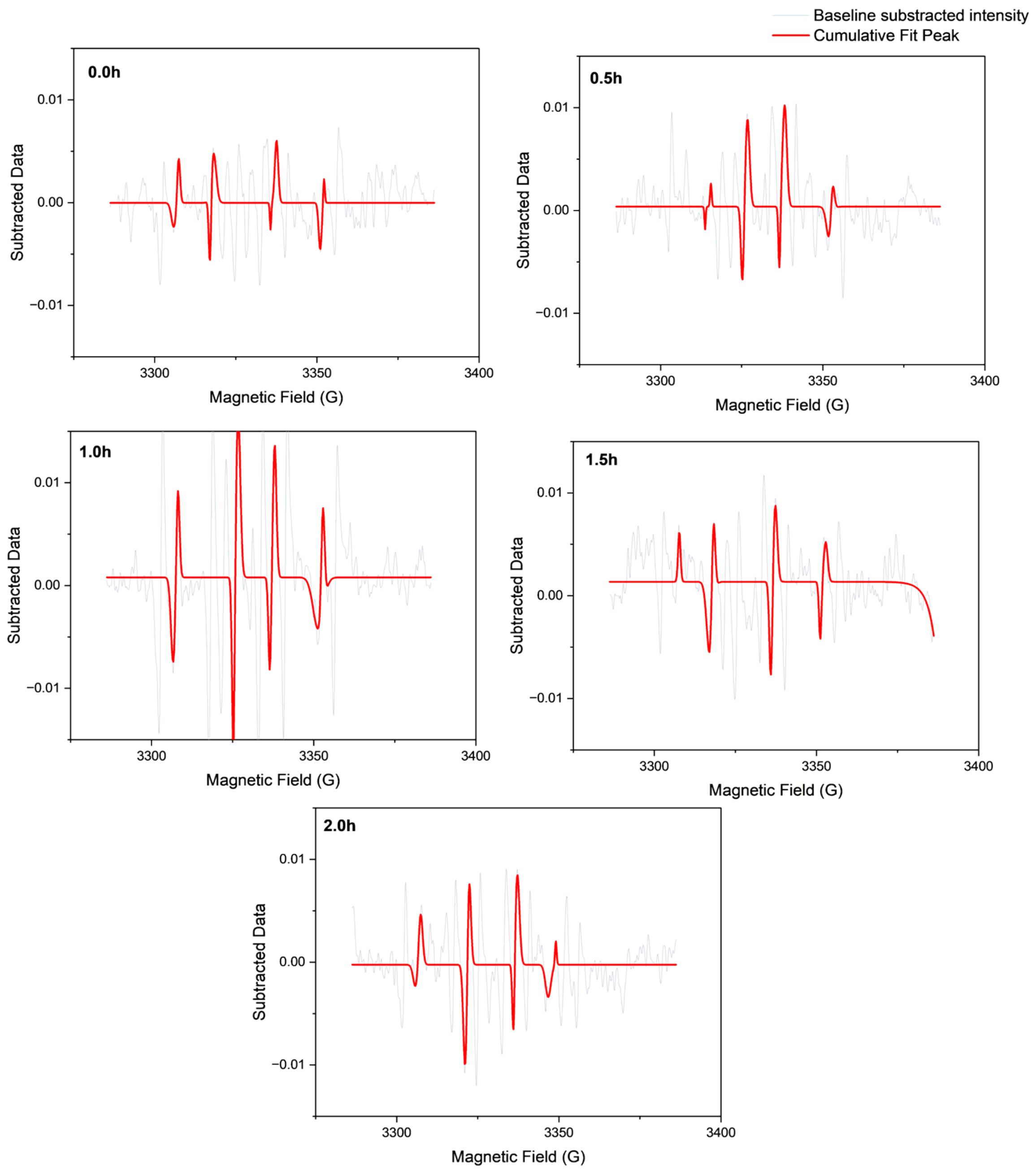

Baseline substracted intensity
Cumulative Fit Peak
0.0h
0.5h
1.0h
1.5h
2.0h
Subtracted Data
Magnetic Field (G)
0.01
0.00
−0.01
3300
3350
3400

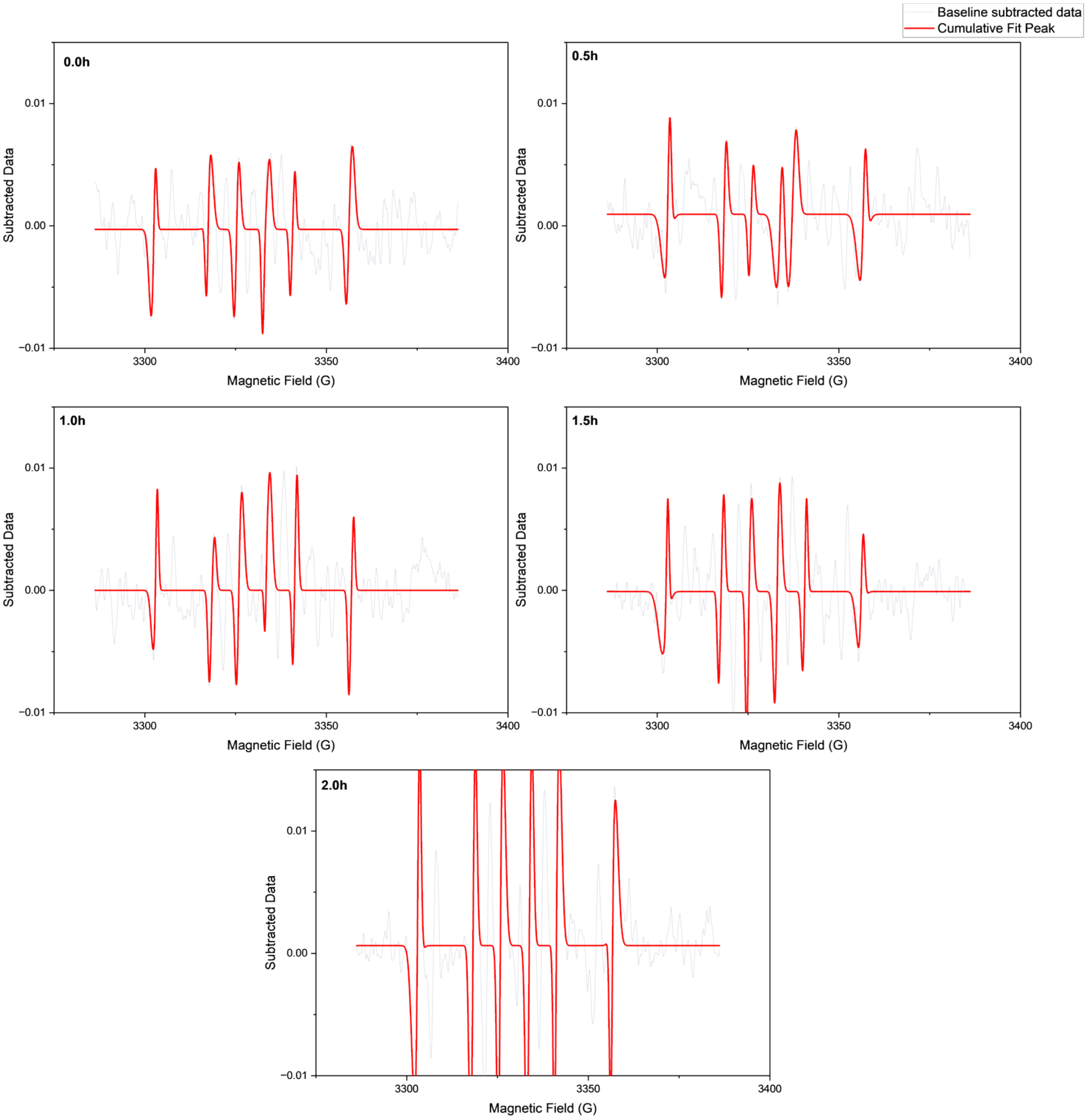

Baseline subtracted data
Cumulative Fit Peak
0.0h
0.5h
1.0h
1.5h
2.0h
Subtracted Data
Magnetic Field (G)
0.01
0.00
−0.01
3300
3350
3400

S10: **AQE reported in other photocatalytic system**

Photocatalytic system	AQE	Reference
Sc-Doped Rutile TiO_2	30%	[12]
Core–shell heterostructure of TiO_2 nanofibers with carbon quantum dots.	52%	[13]
Pt nanoparticle-loaded P25 TiO_2	56%	[14]
PtCu–TiO_2 sandwich photocatalyst	99.2%	[15]

References:

[1] G. V. Buxton, C. L. Greenstock, W. P. Helman, A. B. Ross, "Critical Review of rate constants for reactions of hydrated electrons, hydrogen atoms and hydroxyl radicals (·OH/·O− in Aqueous Solution" *Journal of Physical and Chemical Reference Data* **1988**, *17*, 513–886.

[2] P. Westerhoff, S. P. Mezyk, W. J. Cooper, D. Minakata, "Electron Pulse Radiolysis Determination of Hydroxyl Radical Rate Constants with Suwannee River Fulvic Acid and Other Dissolved Organic Matter Isolates" *Environ. Sci. Technol.* **2007**, *41*, 4640–4646.

[3] T. Arakaki, C. Anastasio, Y. Kuroki, H. Nakajima, K. Okada, Y. Kotani, D. Handa, S. Azechi, T. Kimura, A. Tsuhako, Y. Miyagi, "A General Scavenging Rate Constant for Reaction of Hydroxyl Radical with Organic Carbon in Atmospheric Waters" *Environ. Sci. Technol.* **2013**, *47*, 8196–8203.

[4] J. Hoigné in *Quality and Treatment of Drinking Water II* (Ed.: J. Hrubec), Springer, Berlin, Heidelberg, **1998**, pp. 83–141.

[5] Y. Lee, U. von Gunten, "Oxidative transformation of micropollutants during municipal wastewater treatment: Comparison of kinetic aspects of selective (chlorine, chlorine

dioxide, ferrateVI, and ozone) and non-selective oxidants (hydroxyl radical)" *Water Research* **2010**, *44*, 555–566.

[6], "Xe Arc Lamp - Xenon Lamps," can be found under https://www.newport.com/f/xenon-arc-lamps(accessed 29 November 2024), **n.d.**

[7] K. P. Huber, G. Herzberg, *Molecular Spectra and Molecular Structure*, Springer US, Boston, MA, **1979**.

[8] J. W. Peters, C. S. Foote, "Chemistry of superoxide ion. II. Reaction with hydroperoxides" *J. Am. Chem. Soc.* **1976**, *98*, 873–875.

[9] J. R. Morton, K. F. Preston, "Atomic parameters for paramagnetic resonance data" *Journal of Magnetic Resonance (1969)* **1978**, *30*, 577–582.

[10] Y. Zang, R. Farnood, "Photocatalytic decomposition of methyl *tert*-butyl ether in aqueous slurry of titanium dioxide" *Applied Catalysis B: Environmental* **2005**, *57*, 275–282.

[11] A. S. Hassanien, I. Sharma, "Band-gap engineering, conduction and valence band positions of thermally evaporated amorphous Ge15-x Sbx Se50 Te35 thin films: Influences of Sb upon some optical characterizations and physical parameters" *Journal of Alloys and Compounds* **2019**, *798*, 750–763.

[12] F. Qin, Y. Kang, X. San, Y.-L. Tang, J. Li, X. Zhang, K. Zhang, G. Liu, "Spontaneous Exciton Dissociation in Sc-Doped Rutile TiO2 for Photocatalytic Overall Water Splitting with an Apparent Quantum Yield of 30%" *J. Am. Chem. Soc.* **2025**, *147*, 12897–12907.

[13] J. Cheng, Y. Wang, Y. Xing, M. Shahid, W. Pan, "A stable and highly efficient visible-light photocatalyst of TiO2 and heterogeneous carbon core–shell nanofibers" *RSC Adv.* **2017**, *7*, 15330–15336.

[14] R. Tahawy, M. Esmat, H. El-Hosainy, F. E. Farghaly, E.-S. A. Abdel-Aal, F. I. El-Hosiny, Y. Ide, "A layered titanate nanowire helps Pt/TiO2 photocatalyst for solar hydrogen evolution from water with high quantum efficiency" *bull. Chem. Soc. Jpn.* **2024**, *97*, uoae079.

[15] H. Wang, H. Qi, X. Sun, S. Jia, X. Li, T. J. Miao, L. Xiong, S. Wang, X. Zhang, X. Liu, A. Wang, T. Zhang, W. Huang, J. Tang, "High quantum efficiency of hydrogen production from methanol aqueous solution with PtCu–TiO2 photocatalysts" *Nat. Mater.* **2023**, *22*, 619–626.
